# Commercial Chinese polyherbal preparation: current status and future perspectives

**DOI:** 10.3389/fphar.2024.1404259

**Published:** 2024-07-24

**Authors:** Zhang Chenyao, Hu Haiyin, Shi Menglong, Ma Yucong, Alice Josephine Fauci, Myeong Soo Lee, Wu Xiaolei, Zhang Junhua, Ji Zhaochen

**Affiliations:** ^1^ Evidence-Based Medicine Center, Tianjin University of Traditional Chinese Medicine, Tianjin, China; ^2^ Haihe Laboratory of Modern Chinese Medicine, Tianjin, China; ^3^ Italian National Institute of Health, Rome, Italy; ^4^ Joint Sino-Italian Laboratory of Traditional Chinese Medicine, Italian National Institute of Health, Rome, Italy; ^5^ Korea Institute of Oriental Medicine, Daejeon, Republic of Korea; ^6^ College of Traditional Chinese Medicine, Tianjin University of Traditional Chinese Medicine, Tianjin, China

**Keywords:** traditional Chinese medicine, commercial Chinese polyherbal preparation, Chinese botanical drug, dosage form, ICD-11

## Abstract

**Objective:**

With the modernization of traditional Chinese medicine (TCM) industry, the investment in research and development of new commercial Chinese polyherbal preparations (CCPPs) is increasing, and the varieties of CCPPs are growing. CCPPs play an increasingly important role in the TCM industry. This study has comprehensively summarized and analyzed the current situation of CCPPs that has been on the market in China, and provided suggestions for the research and promotion of CCPPs.

**Methods:**

This study took the CCPPs approved for marketing in domestic drug database of the National Medical Products Administration (NMPA) as the research object, and combined with the publication of related randomized controlled trials (RCTs) of CCPPs in 2020–2022 and the sales of CCPPs in domestic chain pharmacies, statistical analysis was carried out on the drug name, pharmaceutical companies, dosage form, number of flavors, CBDs, ICD-11 classification of diseases treated, etc.

**Results:**

Currently, 58,409 approvals for CCPPs have been issued in China, involving 9,986 varieties of CCPPs, 2,896 pharmaceutical companies and 39 dosage forms. The number of flavors of prescriptions of CCPPs varies from 1 to 90, among which *Glycyrrhiza glabra* L. [Fabaceae; Glycyrrhizae radix et rhizoma] and *Angelica sinensis* (Oliv.) Diels [Apiaceae; Angelicae sinensis radix] are the most widely used. The study found that the CCPPs with the most diverse variety is CCPPs for the treatment of respiratory diseases, some CCPPs can treat multiple system diseases. According to the survey, the sales of CCPPs for respiratory diseases in the chain pharmacies account for more than 1/3 of the total sales of the chain pharmacies, while the number of published randomized controlled trials (RCTs) on CCPPs for circulatory diseases was the largest.

**Conclusion:**

The approval process of CCPPs should be further standardized, and the transformation of TCM prescriptions into CCPPs should be promoted. In the approval process of CCPPs, it is suggested to strengthen the supervision of drug names to clarify the differences between the CCPPs of same name but different prescriptions. Improve the effectiveness and safety of CCPPs by improving the quality of CBDs. It is suggested to optimize the design of new drug research program of CCPPs to avoid waste of research resources.

## 1 Introduction

In China, thousands of years of clinical practice has confirmed that TCM has obvious effects in disease prevention and treatment, healthcare and recuperation, including a variety of intervention measures, such as Chinese medicine, acupuncture and moxibustion, massage, etc., which has played an indispensable role in the process of protecting human health (Liu et al., 2015; Chan et al., 2015; Li et al., 2015). As one of the main intervention measures, TCM has gradually formed a scientific way of compatibility and an industrialized production process in the long process of development, namely, commercial Chinese polyherbal preparation (CCPP)^1^ (Li et al., 2018a). In the narrow sense, CCPP refers to a kind of medicine with certain specifications that can be directly used for disease prevention and treatment after processing or extracting Chinese medicine with a certain formula, such as various pills, powders, granules, etc. The broad sense of CCPP includes not only the narrow sense of CCPP, but also all processed Chinese botanical drugs (CBDs) (NMPA, 2017a). CCPPs have been included in the Chinese Pharmacopoeia and have clear efficacy and indications, covering both prescription and over-the-counter drugs. They are an important metabolite of the Chinese pharmaceutical market. China has vigorously developed the TCM industry and established complete laws and regulations (NATCM, 2016). The Law of the People’s Republic of China on TCM clearly states that “the TCM industry is an important part of China’s medical and health industry” (NATCM, 2016). Compared with TCM decoction, CCPPs have the advantages of stable quality, good curative effect, good safety, fast absorption, convenient taking, carrying and storage (Hsu, 2009; Kang et al., 2019). According to the statistics of the Ministry of Commerce of the People’s Republic of China, in 2021, the sales volume of CCPPs accounts for 14.4% of the total sales volume of seven major categories of medical commodities, and the sales volume of CBDs accounts for 2.2% (MOFCOM, 2022). Currently, CCPPs have a diverse variety and a large market scale. In 2021, CCPPs have a revenue of 486.2 billion yuan, and in the past 3 years, CCPPs will have a profit of 75.52 billion yuan with profit margin 15.53% (The Beijing News, 2023). The Chinese medicine industry is developing rapidly. In addition, the instructions of CCPPs mainly describe the “efficacy” and “indications” about “symptoms”. Compared with the concepts in the instructions of western medicine, it is easier to make independent judgments, and it is easier for non-medical professionals to choose CCPPs in daily life. In order to explore the current situation, relevant research and sales of domestic listed CCPPs, improve the investment distribution of the Chinese medicine industry, and increase the investment in research and development of key CCPPs, and promote CCPPs to enter the international market, we searched and sorted out the existing CCPPs and the RCTs related to CCPPs published in China from 2020 to 2022, analyzed the dosage form, prescription, functions and main indications of CCPPs, and classified them, with a view to providing references for the development of new CCPPs, improvement of prescriptions, and optimization of research design.

## 2 Methods

### 2.1 Data collection

1) With the domestic drug database of the NMPA as the data source, search and enter all CCPPs and corresponding approvals in the NMPA domestic drug database (NMPA, 2023). 2) Retrieve the data included in the clinical evidence database of CCPPs contained in the Evidence Database System (EVDS) of TCM evidence-based research from 2020 to 2022, and extract all RCTs involving CCPPs (TCM Clinical Evidence Database, 2023). 3) Sinohealth provide sales data of all kinds of CCPPs in national chain pharmacies from 2020 to 2022.

### 2.2 Data analysis

**FIGURE 1:**
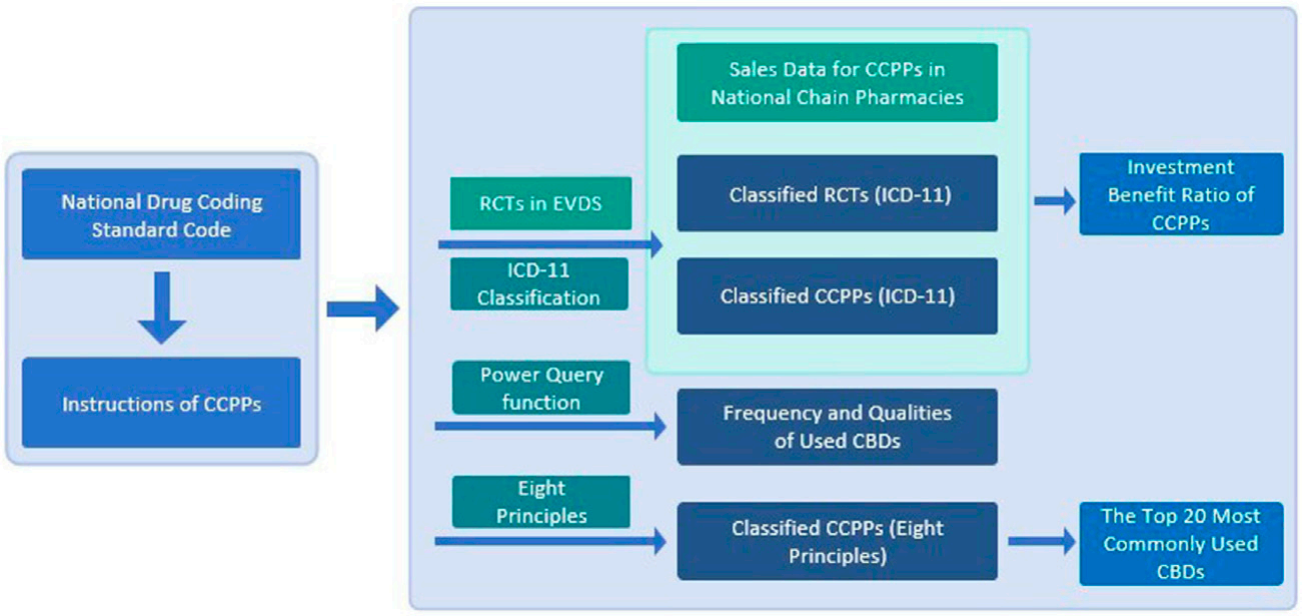
The flowchart for data collection and statistics.

The retrieved data were statistically analyzed, including the name of CCPPs, approval for drugs, pharmaceutical companies, dosage form, number of CBDs, and functional indications. According to the described functions and indications, CCPPs was classified according to the cold and heat, deficiency and excess, and exterior and interior in the eight principles of TCM (except yin and yang), and the diseases treated by CCPPs were classified according to International Classification of Diseases 11th edition (ICD-11). Quantitative analysis was made on dosage form, number of flavors, prescriptions, CCPPs for treatment of diseases of multiple systems, pharmacy sales, publication of RCTs in China and investment benefit ratio. The Power Query of Microsoft EXCEL 2019 and the Origin 2019 were used to conduct statistical analysis. Figure 1 is the flowchart of data collection and statistics. All the botanical drugs were first mentioned in full species name including authorities and family, while only abbreviations when mentioned again. The standardized naming of botanical drugs refers to Medicinal Plant Names Services, an authoritative website that collects the names of plant medicines (Royal Botanic Gardens Kew, 2023). All the botanical drugs follow the ConPhyMP guidelines (Supplementary Material: ConPhyMP checklists Tables 1, 2) (Heinrich et al., 2022).

**TABLE 1:** CCPPs for treatment of 4 or more system diseases.

Drug name	The number of systemic diseases treated	ICD-11 classification for treatment of systemic diseases
Chuanxinlian Capsules (Dropping Pill, Soft Capsule, Dispersible Tablet)	4	No.1, No.12, No.16, No.23
Kushen Capsule (Tablet)	4	No.1, No.13, No.14, No.16
Jinqiancao Capsule (Tablet)	4	No.1, No.13, No.16, No.23
Kangfuxin (Liquid)	4	No.1, No.13, No.18, No.22
Leilong Tablet	4	No.2, No.3, No.12, No.16
Ginseng Polysacchride Injection	4	No.2, No.4, No.5, No.13
Jiedu Jiangzhi Tablet	4	No.5, No.12, No.15, No.21
Compound Garlic Oil Capsule	5	No.1, No.2, No.3, No.11, No.13
Jingtian Sanqi Capsule (Tablet)	5	No.3, No.13, No.15, No.16, No.22
Longdan Xiegan Capsule	6	No.1, No.9, No.10, No.11, No.13, No.16
Palmatine and its different dosage forms (Injection, Tablet, Dispersible Tablet, Capsule, Soft Capsule)	6	No.1, No.9, No.12, No.13, No.16, No.22
Yunnan Hongyao Capsule	6	No.9, No.12, No.13, No.15, No.16, No.22
Qingre Sanjie Capsule (Tablet), Qianliguang Capsule (Tablet)*	7	No.1, No.9, No.10, No.11, No.12, No.13, No.14
Yankening Capsule (Pill, Tablet)	7	No.1, No.9, No.10, No.12, No.13, No.14, No.16
Hemsleyadin	7	No.1, No.9, No.10, No.12, No.13, No.14, No.16
Tanshinone Capsule	7	No.1, No.10, No.12, No.14, No.15, No.16, No.22
Qiangli Fengrujiang Pill	7	No.2, No.3, No.5, No.8, No.11, No.13, No.16

*Note: The main components of Qingre Sanjie Capsule (Tablet) and Qianliguang Capsule (Tablet) are both Qianliguang.

**TABLE 2:** Sales of CCPPs in chain pharmacy in China for diseases of different systems.

ICD-11 classification	Sales volume (yuan)	Percentage (%)
No.12	180,695,572,628.38	37.29
No.21	69,780,529,143.73	14.40
No.13	57,723,242,187.64	11.91
No.11	56,549,579,324.11	11.67
No.15	48,240,234,070.57	9.95
No.16	34,886,361,235.96	7.20
No.14	11,901,180,045.17	2.46
No.8	8,946,488,739.35	1.85
No.9	4,303,759,603.82	0.89
No.2	3,901,660,876.03	0.81
No.5	3,775,105,281.32	0.78
No.10	3,737,038,874.58	0.77
No.3	144,376,730.75	0.03
No.1	14,451,661.13	<0.01
No.4	26,422.77	<0.01

## 3 Results

### 3.1 Approval for CCPPs

The *Measures for the Administration of Drug Registration* issued by the State Administration for Market Regulation stipulate that the format of the approval number for domestically produced drugs is: “national drug approval” and “specific letter” and “four digit year number” and “four digit sequential number” (SAMR, 2020). Therefore, each CCPP on the market has a unique drug approval number, in which “National Drug Approval Z” stands for general Chinese medicine, “National Drug Approval B” stands for healthcare drugs rectified through NMPA, and “National Drug Approval C” stands for CCPPs which comes from ancient classic Chinese traditional medicine metabolites preparations (NMPA, 2002; NMPA, 2020). At present, 58,409 CCPPs have obtained approvals in China, involving 9,986 kinds of CCPPs, and the average number of approval for each CCPP is 5.85; Among them, there are 9,023 kinds of CCPPs have approval of “National Drug Approval Z”, accounting for 90.36%, 958 kinds of CCPPs have approval of “National Drug Approval B”, accounting for 9.59%, and only 5 kinds of CCPPs have approval of “National Drug Approval C”, accounting for 0.05%, which are Xuanfei Baidu Granule, Linggui Zhugan Granule, Sanhan Huashi Granule, Huashi Baidu Granule, and Qingfei Paidu Granule, respectively.

### 3.2 Pharmaceutical companies

**FIGURE 2:**
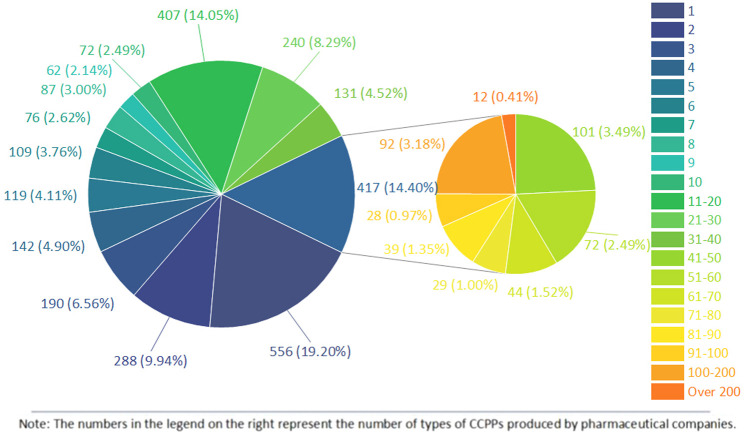
The number of pharmaceutical companies producing certain kinds of CCPPs.

58,409 CCPPs involve a total of 2,896 pharmaceutical companies, of which Tong Ren Tang Pharmaceutical Factory of Beijing Tong Ren Tang Co., Ltd. produces 472 kinds of medicines, followed by Harbin Pharmaceutical Group Shiyitang Co., Ltd. (444), Lanzhou Foci Pharmaceutical Co., Ltd. (417), Beijing Tong Ren Tang Science and Technology Development Co., Ltd. Pharmaceutical Factory (258), and Inner Mongolia Datang Medicine Co., Ltd. (257). The top 10 CCPPs with the most diverse variety approvals are Banlangen Granule (802), Compound Danshen Tablet (647), Liuwei Dihuang Pill (564), Niuhuang Jiedu Tablet (485), Qiju Dihuang Pill (366), Buzhong Yiqi Pill (339), Chuanxinlian Tablet (337), Vitamin C Yinqiao Tablet (320), Shiquan Dabu Pill (319) and Guipi Pill (301). Among 58,409 kinds of CCPPs, 5,575 kinds were produced by only one pharmaceutical company. Among 2,896 companies, 556 pharmaceutical companies only produce one kind of CCPP. Figure 2 shows the number of pharmaceutical companies producing certain kinds of CCPPs.

### 3.3 Dosage form

**FIGURE 3:**
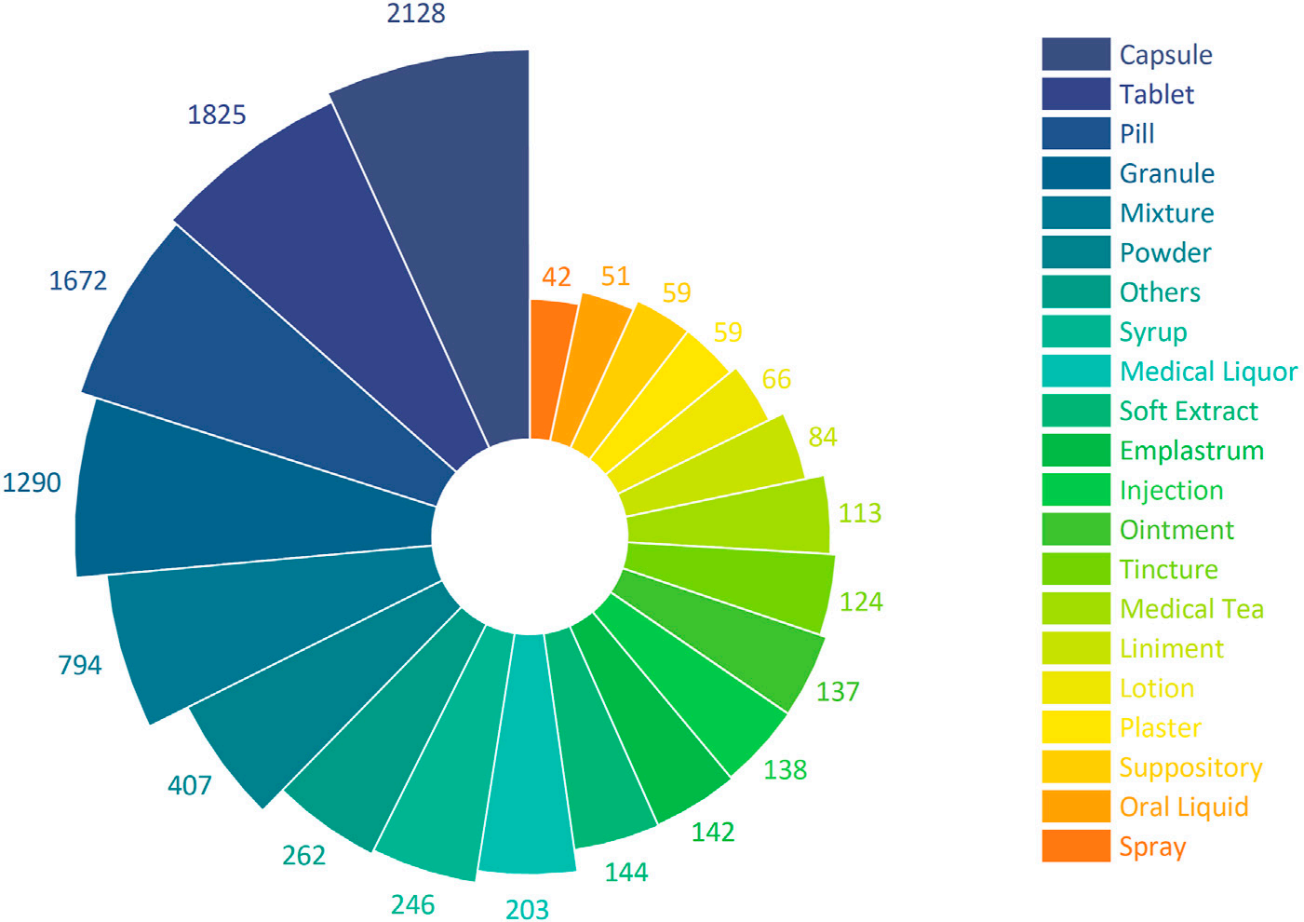
The number of CCPPs with different dosage form.

9,986 CCPPs involve a total of 39 kinds of dosage forms, of which capsule take the most, involving a total of 2,128 CCPPs, followed by tablet (1825), pill (1672), granule (1290), mixture (794), etc. (Figure 3).

### 3.4 Number of flavors

**FIGURE 4:**
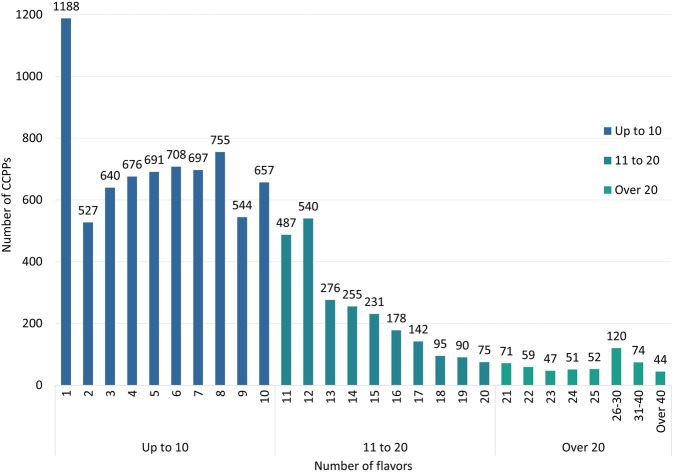
The number of CCPPs of different flavors.

58,409 approvals of CCPPs involve a total of 9,986 kinds of CCPPs. The prescriptions were analyzed according to the instructions. Except for 16 CCPPs that have not reported the composition, the number of flavors of CCPPs ranged from 1 to 90. Among all CCPPs, CCPPs with only one flavor of CBD in the prescription accounts for the highest proportion, a total of 1189 kinds, followed by CCPPs with 8 flavors of CBDs (755), CCPPs with 6 flavors of CBDs (708), CCPPs with 7 flavors of CBDs (697), and CCPPs with 5 flavors of CBDs (691) (Figure 4). There are 7,083 CCPPs’ prescriptions with no more than 10 flavors, and the top five varieties of dosage form are capsule (1,600, 22.59%), tablet (1,418, 20.02%), granule (1,006, 14.20%), pill (834, 11.77%), and mixture (609, 8.60%). There are 2,886 CCPPs’ prescription with more than 10 flavors, and the top five varieties of dosage form are pill (838, 29.04%), capsule (527, 18.26%), tablet (402, 13.93%), granule (284, 9.84%), and mixture (182, 6.31%). With the increase of the number of flavors of CBDs, the proportion of pill in the dosage form has also gradually increased. Among CCPPs with the number of drug flavors less than 10, 10 to 20, 20 to 30 and more than 30, the number and proportion of pill are 834 (11.77%), 641 (27.07%), 174 (36.63%) and 52 (44.07%) respectively. Among the 1,188 CCPPs with only one flavor of CBD, 664 CCPPs have duplicate prescriptions. Taking *Panax notoginseng* (Burkill) F.H.Chen [Araliaceae; Notoginseng radix et rhizoma] as an example, 37 CCPPs take *P. notoginseng* or its extracts as single metabolite, including 17 CCPPs with *P. notoginseng*, 16 CCPPs with *P. notoginseng* saponins, 2 CCPPs with *P. notoginseng* leaf saponins and 2 CCPPs with *P. notoginseng* flower as the main metabolities. Except that 16 CCPPs have not report the main metabolites in the instructions, a total of 2,103 of 9,970 CCPPs had the same name but different prescription, which involve 797 prescriptions. Among them, there are 17 CCPPs with *P. notoginseng* as the main metabolities. In addition to different dosage forms, there are also differences in their indications.

Among them, the efficacy of Shu Sanqi Powder (Tablet) is mainly to replenish blood and improve anemia. Sanqi Mijing Oral Liquid mainly nourishes the heart and clears heat, which can improve symptoms of restlessness and dizziness. Sanqi capsule and Sanqi Guanxinning capsule (dropping pill, tablet) are mainly effective in promoting blood circulation. Sanqi tablet, Sanqi hemostatic capsule, Raw Sanqi powder, Jingtian Sanqi capsule, and Tianqi granule are suitable for various bleeding diseases.

### 3.5 The use of CBDs in the prescription

The eight principles of syndrome differentiation are led by yin and yang, and are comprehensively analyzed based on the patient’s clinical symptoms and signs to explore the nature of the disease, the location of the lesion, the severity of the disease, and the comparison of the strength of the positive and negative sides. They are summarized into eight types of syndromes: yin, yang, exterior, interior, cold, heat, deficiency and excess, which are the basic methods of TCM syndrome differentiation.

According to the indications stated in the instructions, 9,986 CCPPs are classified into cold, heat, exterior, interior, deficiency and excess. It was found that 3,850 (38.55%) CCPPs were used to treat diseases caused by pathogenic heat, 1305 (13.07%) CCPPs were used to treat diseases caused by pathogenic cold, and 4,831 (48.38%) CCPPs were used to treat diseases which have no tendency towards cold or heat. 2,221 (22.24%) CCPPs were used to treat diseases with deficiency syndrome, 6194 (62.03%) CCPPs were used to treat diseases with excess syndrome, and 1,571 (15.73%) CCPPs were used to treat diseases without obvious deficiency or excess syndrome. There are 1,349 (13.51%) CCPPs for diseases with exterior syndrome, 8,427 (84.39%) CCPPs for diseases with interior syndrome, and 210 (2.10%) CCPPs for diseases with exterior and interior syndrome. According to the instructions, the CBDs contained in CCPPs for the treatment of various types of diseases are summarized, and the top 20 CBDs with the highest application frequency are ranked from high to low. The results were shown in Supplementary Tables S1-S3.

The results showed that among the top 20 CBDs in terms of application frequency in CCPPs, except for *Glycyrrhiza glabra* L. [Fabaceae; Glycyrrhizae radix et rhizoma], both CCPPs for the treatment of diseases caused by pathogenic cold and pathogenic heat involve *Angelica sinensis* (Oliv.) Diels [Apiaceae; Angelicae sinensis radix] and *W. cocos* (F.A. Wolf) Ryvarden & Gilb., *A. sinensis*, *W. cocos* and *Citrus reticulata* Blanco [Rutaceae; Citri reticulatae pericarpium] are all involved in CCPPs to treat diseases with excess syndrome and diseases with deficiency syndrome; *C. reticulata* and *Scutellaria baicalensis* Georgi [Lamiaceae; Scutellariae radix] are involved in CCPPs for treating diseases with exterior syndrome and diseases with interior syndrome.

### 3.6 Systematic classification of diseases

**FIGURE 5:**
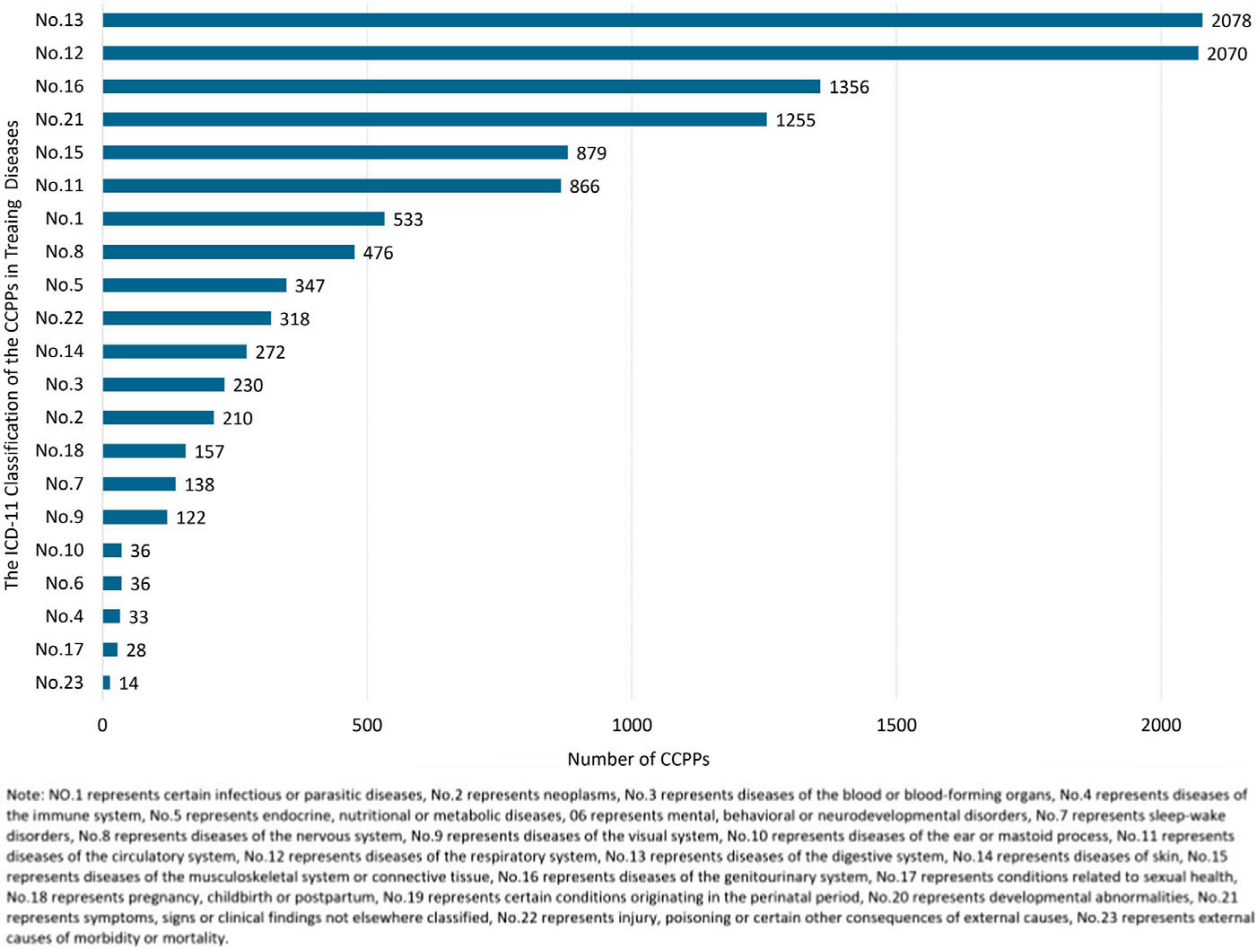
The number of CCPPs in treating different diseases according to ICD-11 classification.

ICD-11 is an international standard for systematically recording, reporting, analyzing, interpreting and comparing health data in a digital way. 9,986 CCPPs were classified referring to the indications described in the instructions of each CCPP combined with ICD-11. It was found that there were 2,078 kinds of CCPPs for the treatment of digestive system diseases, followed by respiratory system diseases (2,070), genitourinary system diseases (1,356), symptoms, signs or clinical findings no elsewhere classified (1,255), musculoskeletal system or connective tissue diseases (879), as shown in Figure 5.

### 3.7 CCPPs for treating multiple system diseases

There are 1,166 CCPPs in 9,986 CCPPs that can treat two or more system diseases, including 941 CCPPs that can treat two system diseases, 191 CCPPs that can treat 3 kinds of systemic diseases, 13 CCPPs that can treat 4 kinds of systemic diseases (Table 1). There are 21 CCPPs that can treat more than 5 kinds of systemic diseases, including Qiangli Fengrujiang Capsule, Hemsleyadin, Compound Garlic Oil Capsule, Yunnan Hongyao Capsule, Tanshinone Capsule, Qingre Sanjie Capsule (Tablet), Longdan Xiegan Capsule, Yankening Capsule (pill, tablet), Palmatine and its different dosage forms (injection, tablet, dispersible tablet, capsule, soft capsule), Jingtian Sanqi Capsule (Tablet).

**FIGURE 6:**
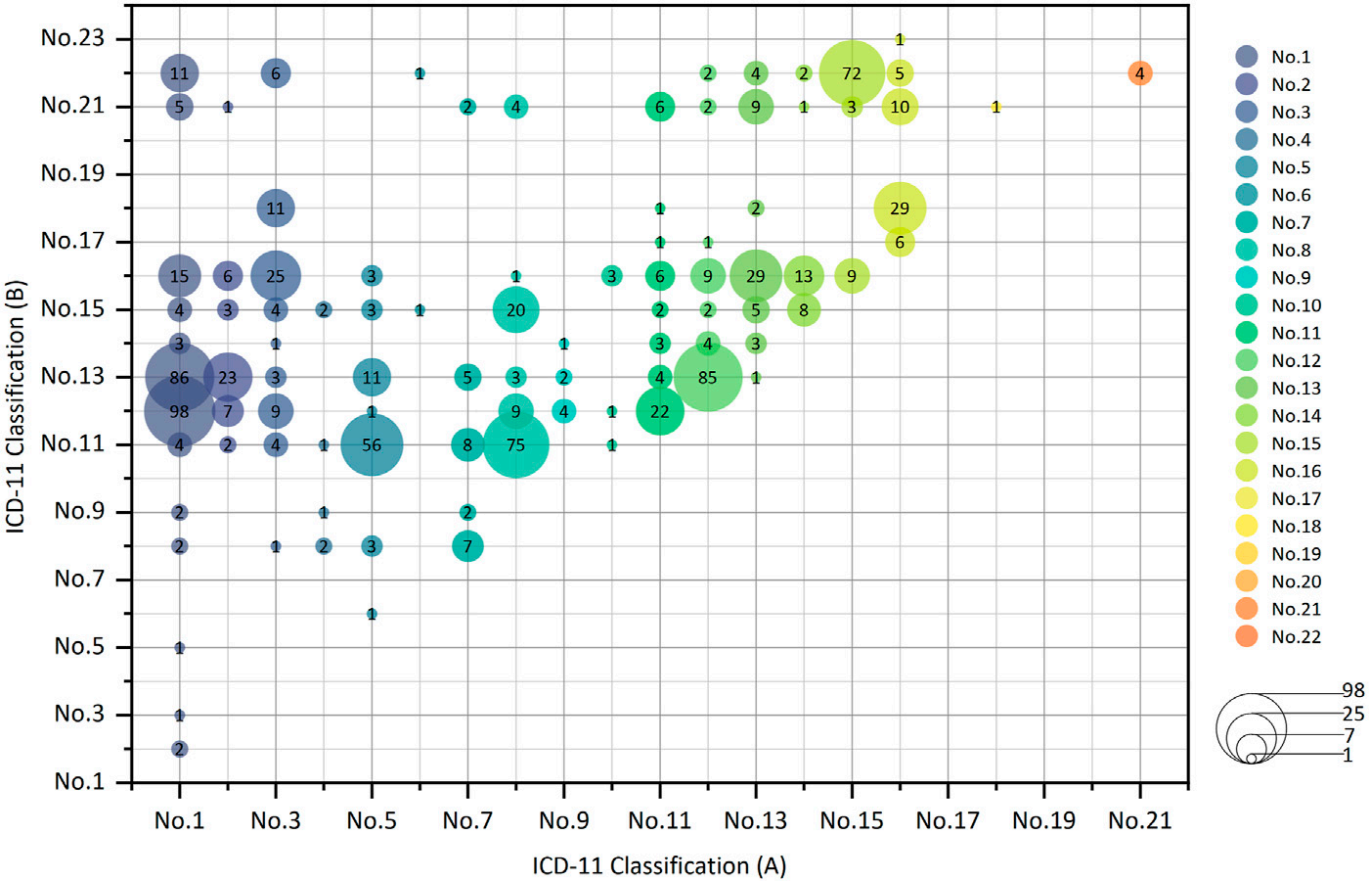
CCPPs treating two kinds of diseases or conditions according to Icd-11 classification.

**FIGURE 7:**
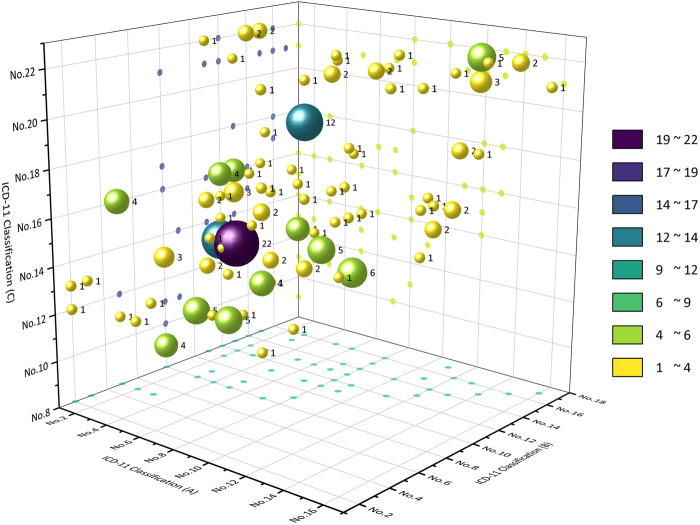
CCPPs treating three kinds of diseases or conditions according to ICD-11 classification.

Among the CCPPs that can treat two kinds of systemic diseases, the top five CCPPs accounted for 44.21%. 98 CCPPs can simultaneously treat certain infectious diseases, parasitic diseases and respiratory diseases (10.41%). 86 CCPPs can simultaneously treat certain infectious diseases, parasitic diseases and digestive diseases (9.14%). 85 CCPPs can simultaneously treat respiratory diseases and digestive diseases (9.03%). 75 CCPPs can simultaneously treat nervous system diseases and circulatory system diseases (7.97%). 72 CCPPs can simultaneously treat musculoskeletal system or connective tissue diseases and some other consequences of injury, poisoning or external causes (7.65%) (Figures 6, 7).

The main metabolite of Chuanxinlian Capsule (Dropping Pill, Soft Capsule, Dispersible Tablet) is *Andrographis paniculata* (Burm.f.) Wall. ex Nees [Acanthaceae; Andrographis herba], and the main active metabolite of andrographis paniculata is andrographolide, which has anti-inflammatory, liver protective, anti-cancer, anti obesity, anti diabetes and other pharmacological activities (Dai et al., 2019; Thakur et al., 2016; Jiang et al., 2021; Mussard et al., 2019; Jadhav and Karuppayil, 2021). The main metabolite of Kushen Capsule (Tablet) is *Sophora flavescens* Aiton [Fabaceae; Sophorae flavescentis radix]. The main active metabolites of sophora flavescens include alkaloids, flavonoids, etc., which have anti allergic, antibacterial, anti-inflammatory, antioxidant, and anti-tumor effects (Wang et al., 2019; Zhong et al., 2017; Cho et al., 2020; Huh et al., 2020; Kan et al., 2020; Jiang et al., 2020). The main metabolite of Jinqiancao Capsule (Tablet) is *Lysimachia christinae* Hance [Primulaceae; Lysimachiae herba], which has pharmacological effects of diuresis, anti stone, anti-inflammatory, and antioxidant (Muanda et al., 2011; Ma et al., 2011; Zhu et al., 2011). The main metabolite of Kangfuxin (Liquid) is the extract of the dried body of Periplanetaamericana, which has anti-tumor, hepatoprotective, antioxidant, antibacterial, and mucosal repair effects (Zhao et al., 2017; Kim et al., 2016; Zhu et al., 2018; Li et al., 2016; Li et al., 2018b; Yun et al., 2017). The main metabolites of Leilong Tablet are *Epimedium brevicornu* Maxim [Berberidaceae; *Epimedii folium*], *Glycine max* (L.). Merr. [Fabaceae; Sojae semen nigrum], *Panax ginseng* C.A.Mey. [Araliaceae; Ginseng radix et rhizoma], *Eleutherococcus senticosus* (Rupr. and Maxim.) Maxim. [Araliaceae; Acanthopanacis senticosi radix et rhizoma seu caulis], *Ophiopogon japonicus* (Thunb.) Ker Gawl. [Asparagaceae; Ophiopogonis radix], *Ziziphus jujuba* Mill. [Rhamnaceae; Jujubae fructus], *G. glabra*, *Aconitum carmichaelii* Debeaux [Ranunculaceae; Aconiti kusnezoffii radix], *Neolitsea cassia* (L.) Kosterm. [Lauraceae; Cinnamomi cortex], *Zingiber officinale* Roscoe [Zingiberaceae; Zingiberis rhizoma recens]*.* The main metabolite of ginseng polysaccharide injection is ginseng polysaccharide, which is an extract from ginseng. It has anti-tumor, treatment of diabetes, antioxidant, liver protection, anti-inflammatory and other effects (Tao et al., 2023; Zhao et al., 2021; Lemmon et al., 2012; Abd et al., 2020). The main metabolite of Jiedu Jiangzhi Tablet is the extract of *Curcuma kwangsiensis* S.G.Lee and C.F.Liang [Zingiberaceae; Polygoni cuspidati rhizoma et radix], which has anti-inflammatory, antiviral, antioxidant, and improving myocardial damage effects (Liu et al., 2018; Zeng et al., 2019; Ding et al., 2014; Lin et al., 2015). The main metabolites of compound garlic oil capsule are concentrated oil of *Allium sativum* L. [Amaryllidaceae; Allii sativi bulbus], gelatin, glycerol, and purified water. Garlic has antibacterial, anti parasitic, antiviral, antioxidant, and improving Alzheimer’s disease effects (Wallock-Richards et al., 2014; Abdel-Hafeez et al., 2015; Gruhlke et al., 2016; Jang et al., 2018; Zhang et al., 2015). The main metabolite of Jingtian Sanqi Tablet and Jingtian Sanqi Capsule is *P*. *notoginseng*, which has hemostatic, anti-inflammatory, anticancer, and antioxidant effects (Xu et al., 2015; Li et al., 2017). The main metabolites of Longdan Xiegan Capsule are *Gentiana scabra* Bunge [Gentianaceae; Gentianae radix et rhizoma], *Bupleurum chinense* DC. [Apiaceae; Bupleuri radix], *S. baicalensis*, *Gardenia jasminoides* J. Ellis [Rubiaceae; Gardeniae fructus], *Alisma plantago-aquatica subsp. orientale* (Sam.) Sam. [Alismataceae; Alismatis rhizoma], *Plantago asiatica* L. [Plantaginaceae; Plantaginis herba], *A. sinensis*, *Rehmannia glutinosa* (Gaertn.) DC. [Orobanchaceae; Rehmanniae radix]*, G. glabra.* The main metabolite of Huangtengsu and its different dosage forms (injection, tablet, dispersible tablet, capsule, soft capsule) is *Fibraurea recisa* Pierre [Menispermaceae; Fibraureae caulis], which has the effects of anti-cancer, improving diabetes, anti-inflammatory, liver protection, improving ischemia reperfusion, etc (Wu et al., 2016; Yue et al., 2017; Zhang et al., 2018; Ma et al., 2016; Lee et al., 2010). The main metabolites of Yunnan Hongyao Capsule are *P*. *notoginseng*, *Paris polyphylla* Sm. [Melanthiaceae; Paridis rhizoma], *Psammosilene tunicoides* W.C.Wu and C.Y.Wu [Caryophyllaceae; Psammosilenes radix], *Aconitum kusnezoffii* Rchb. [Ranunculaceae; Aconiti kusnezoffii radix], *P. tunicoides*, *S. baicalensis*, *Acorus calamus* var. *angustatus* Besser [Acoraceae; Acori tatarinowii rhizoma], etc. The main metabolites of Qianliguang Capsule (Tablet) and Qingre Sanjie Capsule (Tablet) are *Senecio scandens* Buch.-Ham. ex D. Don [Asteraceae; Senecionis scandentis hebra]. Its extracts can anti-inflammatory, antibacterial, anti *leptospira*, liver protection, anti trichomonas, antioxidant, antiviral, anti-tumor, and analgesic effects (Wang et al., 2013). The main metabolites of Yankening Capsule (Tablet, Pill) are *Phellodendron chinense* C.K.Schneid. [Rutaceae; Phellodendri chinensis cortex], *Rheum officinale* Baill. [Polygonaceae; Rhei radix et rhizoma], *S. baicalensis*, *Isatis tinctoria* subsp. tinctoria [Brassicaceae; Isatidis radix], *Coptis chinensis* Franch. [Ranunculaceae; Coptidis rhizoma]. The main metabolite of Xuedansu is *Hemsleya chinensis* Cogn. ex F.B.Forbes & Hemsl. [Cucurbitaceae; hemsleya amobilis Diels], which has pharmacological effects such as anti-tumor, anti-inflammatory, antibacterial, and antiviral (Kim and Choi, 2015; Dharmayanil et al., 2016; Megahed et al., 2020; Singh et al., 2015). The main metabolite of Danshentong Capsule is ethanol extract of *Salvia miltiorrhiza* Bunge [Lamiaceae; Salviae miltiorrhizae radix et rhizoma], which has anti-inflammatory, antioxidant, anti-tumor, and preventive and therapeutic effects on cardiovascular and cerebrovascular diseases (Yang et al., 2023; Wang et al., 2023; Ma et al., 2022; Zhou et al., 2021; Lan et al., 2022). The main metabolites of the Qiangli Fengrujiang Pill are royal jelly, *P. ginseng*, gelatin, etc.

### 3.8 National chain pharmacy sales

According to the sales data of national chain pharmacy provided by Sinohealth, it is found that among the CCPPs sold on the market from 2020 to 2022, the top 10 CCPPs in the pharmacy sales are CCPPs for respiratory system disease (180.70 billion yuan, 37.29%), nourishing and healthcare CCPPs (69.78 billion yuan, 14.40%), CCPPs for digestive system disease (57.72 billion yuan, 11.91%), CCPPs for circulatory system disease (56.55 billion yuan, 11.67%), CCPPs for diseases of musculoskeletal system or connective tissue (48.24 billion yuan, 9.95%), CCPPs for diseases of the genitourinary system (34.89 billion yuan, 7.20%), CCPPs for diseases of skin (11.90 billion yuan, 2.46%), CCPPs for diseases of nervous system (8.95 billion yuan, 1.85%), CCPPs for diseases of visual system (4.30 billion yuan, 0.89%), and medications for tumor treatment (3.90 billion yuan, 0.81%) (Table 2).

### 3.9 Publication of RCTs of CCPPs in the treatment of diseases

Based on the data contained in the clinical evidence database of CCPPs contained in EVDS of Chinese medicine evidence-based research, all published RCTs of CCPPs in treating diseases in China from 2020 to 2022 were counted, with a total of 5,738. According to the ICD-11 classification, it was found that the top 10 studies focus on circulatory system diseases (1030,17.95%), respiratory system diseases (862,15.02%), genitourinary system diseases (792,13.80%), digestive system diseases (664,11.57%), nervous system diseases (599,10.44%), endocrine, nutritional or metabolic diseases (331,5.77%), infectious or parasitic diseases (204,3.56%), skin diseases (185,3.22%), neoplasms (147,2.56%), and symptoms, signs or clinical findings not elsewhere classified (136,2.37%) (Table 3).

**TABLE 3:** Publication of RCTs of CCPPs in the treatment of diseases.

ICD-11 classification	Counts	Percentage (%)
No.11	1,030	17.95
No.12	862	15.02
No.16	792	13.80
No.13	664	11.57
No.8	599	10.44
No.5	331	5.77
No.15	229	3.99
No.1	204	3.56
No.14	185	3.22
No.2	147	2.56
No.21	136	2.37
No.6	121	2.11
No.22	114	1.99
No.18	108	1.88
No.9	56	0.98
No.10	37	0.64
No.3	34	0.59
No.7	28	0.49
No.19	24	0.42
No.4	14	0.24
No.24	13	0.23
No.23	5	0.09
No.17	5	0.09

### 3.10 Investment benefit ratio of CCPPs

**FIGURE 8:**
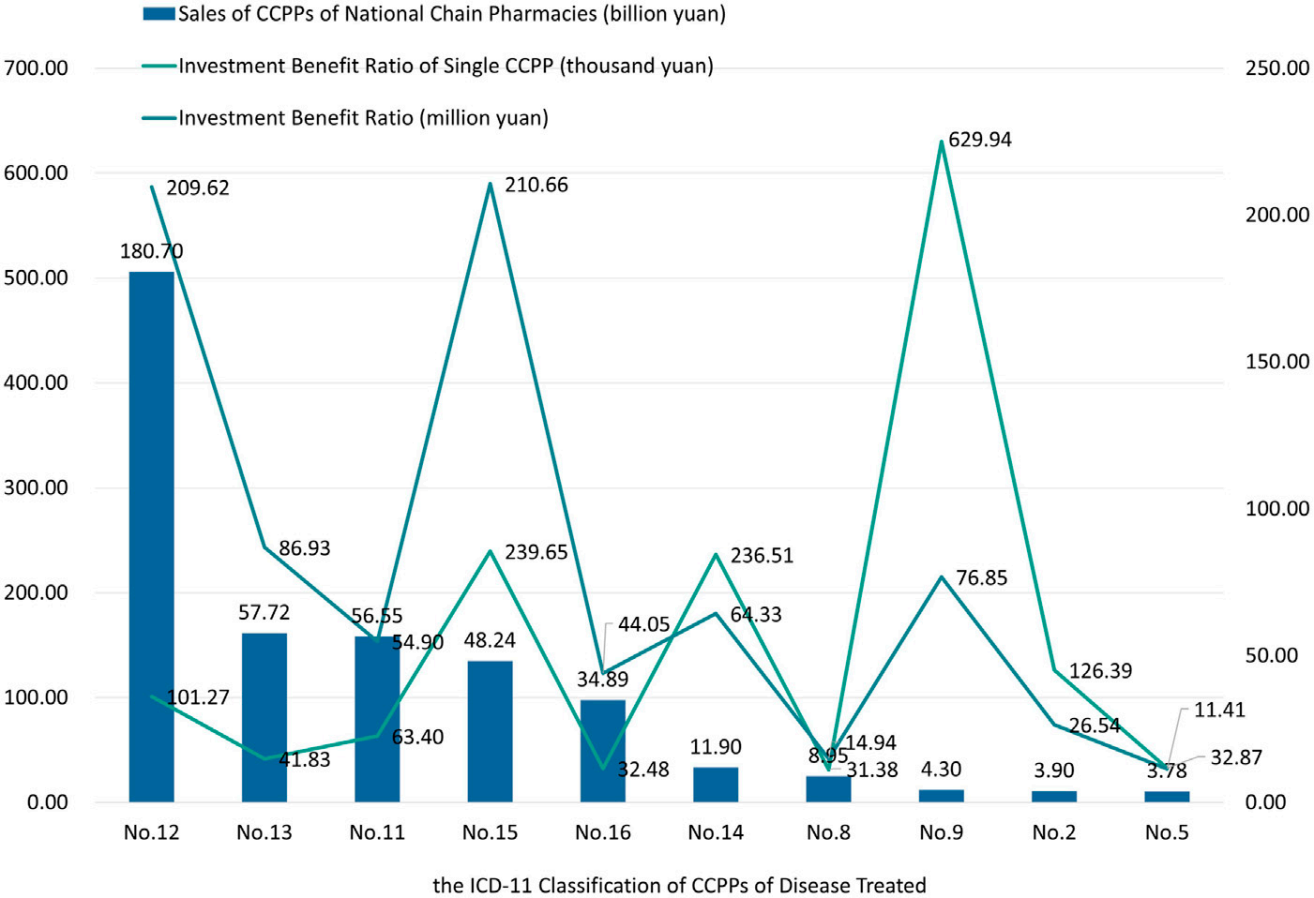
The investment benefit ratio and investment benefit ratio of single CCPP of top 10 sales CCPPs.

Compare the number of CCPPs ranking top 10 in terms of sales, the number of CCPPs and the number of RCTs published, and calculate the investment benefit ratio (sales volume/number of RCTs published) of CCPPs in each ICD-11 classification and the investment benefit ratio of single CCPP. The results are shown in Figure 8. Among them, CCPPs for the treatment of musculoskeletal system or connective tissue diseases has the highest investment benefit ratio of 210.66 million yuan, and its single CCPP investment benefit ratio is 239.65 thousand yuan. The investment benefit ratio of CCPPs for the treatment of respiratory diseases ranks second, with 209.62 million yuan, which was close to CCPPs for the treatment of musculoskeletal system or connective tissue diseases. However, because there were 2070 kinds of CCPPs for respiratory diseases, the investment benefit ratio of single CCPP was only 101.27 thousand yuan. The market sales of CCPPs for visual system diseases is only 4.30 billion yuan, with an investment benefit ratio of 76.85 billion yuan. However, due to the small number of drugs, the single CCPPs has the highest investment benefit ratio of 629.94 thousand yuan. There are 2,078 kinds of CCPPs used for digestive system diseases, but the investment benefit ratio ranks third with 86.93 million yuan. The investment benefit ratio of a single CCPP is only 41.83 thousand yuan, far lower than that of CCPPs used for respiratory system diseases.

## 4 Discussion

### 4.1 Approval for CCPPs

At present, the approval number of CCPPs on the market include CCPPs with “National Drug Approval Z”, some healthcare products with “National Drug Approval B”, and the ancient classic Chinese medicine compound preparations with “National Drug Approval C”. The CCPPs with the approval number of “National Drug Approval B” are TCM healthcare products with therapeutic effect. In August 1995, the China’s Ministry of Health issued a notice on strengthening the supervision and management of drug approval numbers, requiring the health administrative departments of each province to clean up and rectify approved health products. The production and sales of health drugs with therapeutic effect should be stopped, and new drug approval procedures should be followed. Among them, TCM health drugs should be approved according to regulations. In March 2000, NMPA issued a notice on the rectification of TCM health drugs, requiring the re issuance of approval numbers for Chinese medicine health products that meet the requirements after rectification (NMPA, 2017b). In January 2002, NMPA issued a notice on the unified replacement and standardization of drug approval number formats, which stipulated the use of the letter “B” for healthcare drugs that had been rectified by NMPA (NMPA, 2000).

Most of the CCPPs on the market have the approval number of “National Drug Approval Z”. This kind of CCPPs and “National Drug Approval B” belong to TCM, both of which have therapeutic effects and were released in January 2002. However, the approval process for drugs with “National Drug Approval Letter B” is relatively simple compared to “National Drug Approval Letter Z” (NMPA, 2024e, NMPA, 2024h). The standards for clinical trials, pharmacological and toxicological testing data are relatively loose, and there is no mandatory requirement for on-site verification of clinical trials or providing original clinical trial records. Therefore, although these health products have successfully transformed into CCPPs, their safety and efficacy standards are not as clear as those of “National Drug Approval Letter Z” drugs, which also creates many uncertain factors for their inclusion in the domestic medical insurance drug catalog and international promotion.

In order to promote the research and industrial development of new TCM, the country issued the approval number of “National Drug Approval C” in 2020. This kind of approval number of CCPPs uses traditional preparation processes and traditional routes of administration, and its main functions are expressed in Chinese medicine terms. Compared with other types of CCPPs, this type of CCPPs does not need a phase III clinical trial, and because this type of CCPPs has developed a compound preparation from the ancient classic famous formula, it has gone through long-term clinical practice, and its safety and effectiveness have been guaranteed.

The classification of approval numbers of CCPPs only indicates that the requirements for registration and application materials of different approval numbers are different, which does not represent the level of drug development and drug efficacy. In the future, more CCPPs in the category of “National Drug Approval C” will be released. We suggest the introduction of new guidance documents to improve the approval standards for “National Drug Approval B” drugs and accelerate the transition to “National Drug Approval Z” drugs.

### 4.2 Name of CCPPs

At present, there are a large number of CCPPs with same prescription but different name. For example, there are 7 kinds of CCPPs with ligusticum wallichii and rhizoma gastrodiae as the main metabolites, namely, Dachuanxiong Oral Liquid (granule, tablet, capsule) and Tianshu Capsule (tablet, soft capsule, dropping pill). A large number of CCPPs with same prescription but different name circulate in the market, which may lead to difficulties for the majority of patients in selecting medicines, and also lay hidden dangers for the unified production, processing and formulation of CCPPs. For example, the main metabolites of Huoxue Tongmai (HXTM) Tablet are *Spatholobus suberectus* Dunn [Fabaceae; Spatholobi caulis], *Prunus persica* (L.) Batsch [Rosaceae; Persicae semen], *S. miltiorrhiza*, *Paeonia lactiflora* Pall. [Paeoniaceae; Paeoniae radix rubra], *Carthamus tinctorius* L. [Asteraceae Carthami flos], *Dalbergia odorifera* T.C.Chen [Fabaceae; Dalbergiae odoriferae lignum], *Curcuma longa* L. [Zingiberaceae; Curcumae longae rhizoma], *P. notoginseng*, *Ligusticum chuanxiong* Hort. [Apiaceae; Chuanxiong rhizoma], *C. reticulata*, *Dolomiaea costus* (Falc.) Kasana and A. K. Pandey [Asteraceae; Aucklandiae radix], *A. calamus*, *Lycium chinense* Mill. [Solanaceae; Lycii fructus], *Polygonatum kingianum* Collett & Hemsl. [Asparagaceae; Polygonati rhizoma], *P. ginseng*, *O. japonicus*, Borneolum syntheticum. The instruction mentions that HXTM Tablet has the effects of promoting blood circulation and relieving pain, and is commonly used in clinical practice for angina caused by coronary atherosclerosis. By shrinking the plaque, reducing plaque thickness, ameliorating carotid blood flow, decreasing degree of platelet activation and anti-inflammatory response to make plaques stable (Huang et al., 2017). HXTM Capsule only includes Whitmania pigra Whitman [Hirudo], which has the effects of activating blood, resolving stasis, dredging meridians and relieving pain. HXTM Capsule can be used to treat blood stasis, amenorrhea, and injuries. The two drugs share the same name of HXTM but there is a significant difference in metabolites and therapeutic effects, which is easily confused for ordinary patients who do not have pharmaceutical knowledge, even if clinicians they may also not know very clearly.

According to the Technical Guidelines for the Naming of General Names of CCPPs, the names of CCPPs, except for dosage forms, should not be repeated with the existing common names of CCPPs (NMPA, 2017b). Therefore, it is suggested to strengthen the supervision of drug names in the approval process of CCPPs, fully report the processing methods of medicinal materials, compatibility doses and other ways, and clarify the differences between CCPPs with the same prescription, so as to reduce the occurrence of CCPPs with same prescription but different name.

### 4.3 The use of CBDs in the prescription

In the instructions of CCPPs, there are a large number of CBDs with unclear processing methods, such as rehmannia glutinosa and liquorice, which may lead to insufficient clinical application accuracy of CCPPs. In addition, some CBDs’ names are not expressed uniformly. For example, honeysuckle is sometimes referred to as rendong, rather than jinyinhua, which brings certain difficulties to the standardization of the instructions. Few CCPPs have specific dosage in their instructions, and some CBDs also have specific drug application part. It is suggested that the processing method and dosage of CBDs should be clearly reported in the instructions of CCPPs, and unified names for CBDs, so as to improve the accuracy of the application of the formula.

At present, there are problems in the production and processing of CBDs, such as sulfur addition, pesticide residue, aflatoxin pollution, and excessive heavy metal content. Therefore, for large amounts of CBDs, such as *G. glabra*, *A*. *sinensis*, *W. cocos*, etc., it is necessary to clearly define harmful substance detection standards, strengthen supervision in the processing, storage, and circulation of CBDs, continuously improve the quality, and standardize production processes, In order to ensure the efficacy and safety of CCPPs.

### 4.4 Multi-metabolites CCPPs

At present, among the prescription of CCPPs for the treatment of multiple system diseases, there may be some differences between the prescriptions of CCPPs with same name from different pharmaceutical factories, the content of each Chinese medicine, and the processing method, which leads to more pharmacological research on CCPPs with single metabolite, but relatively less research on multi-metabolites CCPPs, which leads to the inability to combine research on multi-metabolites CCPPs of the same kind, resulting in a waste of research resources. CCPPs with the same prescription should be named uniformly, the requirements for CCPPs with the same name but different dosage forms should be standardized, and the supervision of CCPPs with the same name but different prescriptions and CCPPs with the same name but different dosage forms should be strengthened. The processing method and dosage of CBDs shall be clearly reported to standardize the contents of the instructions of CCPPs. The specification of CCPPs instructions is to improve drug quality and guide clinical rational drug use. The quality control of CCPPs should achieve a full chain and all-round supervision, including the planting, processing and storage of CCPPs, as well as the quality and efficacy evaluation of CCPPs. The frequent abuse of pesticides in the current cultivation process of traditional Chinese medicine seriously affects the quality of drugs. Strictly control pesticide use standards, keep records of pesticide use, include the ‘sulfur free processing, no aflatoxin, no highly toxic pesticides and full traceability’ brand standards into the rigid evaluation indicators of the Chinese medicinal material industry as appropriate, and destroy the substandard and unqualified CBDs to avoid entering the CCPPs processing market. At present, the market of CBDs is often characterized by mixed authenticity and shoddy products. Therefore, it is necessary to establish an industry standard for quality testing of CBDs, introduce quality markers into the process of quality evaluation of medicinal materials, and require CCPPs manufacturers to clearly state the grade of CBDs used. At present, NMPA has released the National Standard for the Processing of CBDs and will officially implement it by the end of 2023. However, this standard does not include all CBDs, and the current instructions do not require the preparation method to be specified. It is recommended to gradually supplement and improve this preparation standard, and require the instructions to standardize the preparation method of CBDs.

CCPPs flexibly adapts to the treatment of multiple systemic diseases, fully reflecting the characteristic diagnosis and treatment thinking of TCM in treating diseases from syndrome and symptom.

### 4.5 Investment benefit ratio of CCPPs

Based on the research results, it can be seen that CCPPs for respiratory diseases have the most diverse variety, and they also have the largest sales volume and the highest market share. However, the investment benefit ratio of single CCPP is relatively low. In contrast, CCPPs for visual system diseases have fewer varieties, and the sales volume only account for 0.89% of the total, the investment benefit ratio is positively correlated. The investment benefit ratio of single CCPP is relatively high, which means the market needs further exploration.

In contrast, CCPPs for digestive system diseases accounted for 11.91% of the market sales, but their variety takes the most. Therefore, although the investment benefit ratio was similar to that of CCPPs for visual system diseases, the investment benefit ratio of single CCPP is far lower than that of CCPPs for visual system diseases. In addition, sales of CCPPs for digestive diseases is less than one-third of CCPPs for respiratory diseases, but their number of CCPPs are similar. It can be inferred that there is a surplus of CCPPs on the market to treat respiratory diseases and digestive diseases. Competition among different CCPPs is fierce, and the investment benefit ratio of pharmaceutical companies is significantly reduced. It is suggested that pharmaceutical enterprises further optimize the design of new CCPP research and development programs to avoid waste of research resources. It is suggested to promote secondary development of head CCPPs, expand product manuals, explore new medication scenarios, and reduce resource waste.

## 5 Conclusion

At present, there is no published review on the status of CCPPs. By analyzing the status of CCPPs approved on the market in the domestic drug database of the NMPA, and combining the publication of related RCTs of CCPPs from 2020 to 2022 and the sales of CCPPs in domestic chain pharmacies, the name, pharmaceutical companies, dosage form, number of flavors, CBDs, classification of treated diseases according to ICD-11, indications of CCPPs are summarized and analyzed. In the future, it is suggested to further standardize the approval process of CCPPs, strengthen the supervision of drug names, fully report the processing methods and compatibility doses of CCPPs, clarify the differences between the CCPPs with same name, standardize the contents of the instructions, improve the quality of CCPPs, reasonably design the compatibility of new drugs, so as to further improve the effectiveness and safety of CCPPs, and reduce the waste of research resources. This study also has some limitations. Some CCPPs formulations are confidential, so they cannot be included in this statistics. Since most CCPPs’ instructions do not specify the amount of CBDs, this article only counts the types of CBDs involved. With the improvement of the instructions, research on the amount of CBDs used in CCPPs prescriptions should be supplemented to provide reference for planting. In addition, the sales and RCTs data for 2023 had not been issued yet, so only the sales of CCPPs in 2020–2022 were counted. The sales of CCPPs for respiratory diseases may be relatively increased due to the impact of the COVID-19. Therefore, future research should continue to pay attention to the standardization of instructions. For RCTs included in this article, statistical analysis of relevant intervention measures and outcomes should be supplemented later to clarify the efficacy advantages of CCPPs.

## Data Availability

Publicly available datasets were analyzed in this study. This data can be found here: https://www.nmpa.gov.cn/zwfw/zwfwzxfw/zxfwsjxz/20230706145013131.html.

## Footnotes

1 Note: In the 19th century, patent medicine referred to ill-defined and unregulated drug, which the manufacture claimed to cure or prevent nearly every ailment but actually with questionable effectiveness, even have potential for harmful side effects (Digital Public Library of America, 2019; Haglery Museum, 2017; Wikipedia, 2024). Therefore instead of Chinese patent medicine, we choose CCPP as a more appropriate expression.

## References

[B1] Abd E. M. Abd E. L. A. Hassan A. EL-Borai N. B. (2020). Ginseng attenuates fipronil-induced hepatorenal toxicity via its antioxidant, anti-apoptotic, and anti-inflammatory activities in rats. Environ. Sci. Pollut. Res. Int. 27 (36), 45008–45017. 10.1007/s11356-020-10306-0 32772290

[B2] Abdel-Hafeez E. H. Ahmad A. K. Kamal A. M. Abdellati M. Z. Abdelgelilf N. H. (2015). *In vivo* antiprotozoan effects of garlic (Allium sativum) and ginger (Zingiber oicinale) extracts on experimentally infected mice with Blastocystis spp. Parasitol. Res. 114 (9), 3439–3444. 10.1007/s00436-015-4569-x 26085068

[B3] Chan K. Zhang H. Lin Z. X. (2015). An overview on adverse drug reactions to traditional Chinese medicines. Br. J. Clin. Pharmacol. 80 (4), 834–843. 10.1111/bcp.12598 25619530 PMC4594726

[B4] Cho B. O. Che D. N. Kim J. S. Kim J. H. Shin J. Y. Kang H. J. (2020). *In vitro* Anti-Inflammatory and Anti-Oxidative Stress Activities of Kushenol C Isolated from the Roots of Sophora flavescens. Molecules 25 (8), 1768. 10.3390/molecules25081768 32290603 PMC7221590

[B5] Dai Y. Chen S. R. Chai L. Zhao J. Wang Y. Wang Y. (2019). Overview of pharmacological activities of Andrographis paniculata and its major compound andrographolide. Crit. Rev. Food Sci. Nutr. 59 (Suppl. 1), S17–S29. 10.1080/10408398.2018.1501657 30040451

[B6] Dharmayanil N. Juliawaty L. D. Syaha Y. M. (2016). Three Tetracyclic Triterpenoic Acids from Dysoxylum densiflorum and Their Antibacterial Activities. Nat. Prod. Commun. 11 (8), 1081–1083. 10.1177/1934578x1601100812 30725562

[B7] Digital Public Library of America (2019). Patent medicine, 1860-1920. Digital Public Library of America. Available at: https://dp.la/exhibitions/patent-medicine/1860-1920?item=1290.

[B8] Ding W. Dong M. Deng J. Yan D. Liu Y. Xu T. (2014). Polydatin attenuates cardiac hypertrophy through modulation of cardiac Ca2+ handling and calcineurin-NFAT signaling pathwa. Am. J. Physiol. Heart Circ. Physiol. 307 (5), H792–H802. 10.1152/ajpheart.00017.2014 25015961

[B9] Gruhlke M. C. Nicco C. Batteux F. Slusarenko A. J. (2016). The Effects of Allicin, a Reactive Sulfur Species from Garlic, on a Selection of Mammalian Cell Lines. Antioxidants (Basel) 6 (1), 1. 10.3390/antiox6010001 28035949 PMC5384165

[B10] Haglery Museum (2017). History of Patent Medicine. Haglery Museum. Available at: https://www.hagley.org/research/digital-exhibits/history-patent-medicine (Accessed: 2024).

[B11] Heinrich M. Jalil B. Abdel-Tawab M. Echeverria J. Kulic Z. Mcgaw L. J. (2022). Best Practice in the chemical characterisation of extracts used in pharmacological and toxicological research-The ConPhyMP-Guidelines. Front. Pharmacol. 13, 953205. 10.3389/phar.2022.953205 36176427 PMC9514875

[B12] Hsu E. (2009). Chinese propriety medicines: an “alternative modernity?” The case of the anti-malarial substance artemisinin in east africa. Med. Anthropol. 28 (2), 111–140. 10.1080/01459740902848303 19404880

[B13] Huang Qunlian X. S. H. Y. (2017). Intervention of Huoxue Tongmai Tablets on Carotid Vulnerable Plaques. Chin. J. Exp. Traditional Med. ormulae 23 (08), 184–189.

[B14] Huh J. W. Lee J. H. Jeon E. Ryu H. W. Oh S. R. Ahn K. S. (2020). Maackiain, a compound derived from Sophora flavescens, increases IL-1beta production by amplifying nigericin-mediated inflammasome activation. EBS Open Bio 10 (8), 1482–1491. 10.1002/2211-5463.12899 PMC7396426 32428336

[B15] Jadhav A. K. Karuppayil S. M. (2021). Andrographis paniculata (Burm. F) Wall ex Nees: Antiviral properties. Phytother. Res. 35 (10), 5365–5373. 10.1002/ptr.7145 33929758

[B16] Jang H. J. Lee H. J. Yoon D. K. Ji D. S. Kim J. H. Lee C. H. (2018). Antioxidant and antimicrobial activities of fresh garlic and aged garlic by-products extracted with different solvents. Food Sci. Biotechnol. 27 (1), 219–225. 10.1007/s10068-017-0246-4 30263743 PMC6049750

[B17] Jiang M. Sheng F. Zhang Z. Ma X. Gao T. Fu C. (2021). Andrographis paniculata (Burm.f.) Nees and its major constituent andrographolide as potential antiviral agents. J. Ethnopharmacol. 272, 113954. 10.1016/j.jep.2021.113954 33610706

[B18] Jiang P. Sun Y. Cheng N. (2020). Liver metabolomic characterization of Sophora flavescens alcohol extract-induced hepatotoxicity in rats through UPLC/LTQ-Orbitrap mass spectrometry. Xenobiotica 50 (6), 670–676. 10.1080/00498254.2019.1687962 31747812

[B19] Kan L. L. Liu D. Chan B. C. Tsang M. S. Hou T. Leung P. C. (2020). The flavonoids of Sophora flavescens exerts anti-inflammatory activity via promoting autophagy of Bacillus Calmette-Guerin-stimulated macrophages. J. Leukoc. Biol. 108 (5), 1615–1629. 10.1002/JLB.3MA0720-682RR 32794339

[B20] Kang T. Dou D. Xu L. (2019). Establishment of a quality marker (Q-marker) system for Chinese herbal medicines using burdock as an example. Phytomedicine 54, 339–346. 10.1016/j.phymed.2018.04.005 30318153

[B21] Kim E. K. Choi E. J. (2015). Compromised MAPK signaling in human diseases: an update. Arch. Toxicol. 89 (6), 867–882. 10.1007/s00204-015-1472-2 25690731

[B22] Kim I. W. Lee J. H. Subramaniyam S. Yun E. Y. Kim I. Park J. (2016). De Novo Transcriptome Analysis and Detection of Antimicrobial Peptides of the American Cockroach Periplaneta americana (Linnaeus). PLoS One 11 (5), e0155304. 10.1371/journal.pone.0155304 27167617 PMC4864078

[B23] Lan J. Li K. Gresham A. Miao J. (2022). Tanshinone IIA sodium sulfonate attenuates inflammation by upregulating circ-Sirt1 and inhibiting the entry of NF-kappaB into the nucleus. Eur. J. Pharmacol. 914, 174693. 34896110 10.1016/j.ejphar.2021.174693

[B24] Lee W. C. Kim J. K. Kang J. W. Oh W. Y. Jung J. Y. Kim Y. S. (2010). Palmatine attenuates D-galactosamine/lipopolysaccharide-induced fulminant hepatic failure in mice. Food Chem. Toxicol. 48 (1), 222–228. 10.1016/j.ct.2009.10.004 19818826

[B25] Lemmon H. R. Sham J. Chau L. A. Madrenas J. (2012). High molecular weight polysaccharides are key immunomodulators in North American ginseng extracts: characterization of the ginseng genetic signature in primary human immune cells. J. Ethnopharmacol. 142 (1), 1–13. 10.1016/j.jep.2012.04.004 22521964

[B26] Li D. Li W. Chen Y. Liu L. Ma D. Wang H. (2018a). Anti-fibrotic role and mechanism of Periplaneta americana extracts in CCl4-induced hepatic fibrosis in rats. Acta Biochim. Biophys. Sin. (Shanghai) 50 (5), 491–498. 10.1093/abbs/gmy024 29538616 PMC5946930

[B27] Li H. Deng J. Yue Z. Zhang Y. Sun H. (2015). Detecting drug-herbal interaction using a spontaneous reporting system database: an example with benzylpenicillin and qingkailing injection. Eur. J. Clin. Pharmacol. 71 (9), 1139–1145. 10.1007/s00228-015-1898-8 26159784

[B28] Li H. Wang S. Yue Z. Ren X. Xia J. (2018b). Traditional Chinese herbal injection: current status and future perspectives. Fitoterapia 129, 249–256. 10.1016/j.itote.2018.07.009 30059719

[B29] Li M. Qi Z. Hao Y. Lv C. Jia L. Wang J. (2017). New Adducts of Iriflophene and Flavonoids Isolated from Sedum aizoon L. with Potential Antitumor Activity. Molecules 22 (11), 1859. 10.3390/molecules22111859 29099046 PMC6150161

[B30] Lin C. J. Lin H. J. Chen T. H. Hsu Y. A. Liu C. S. Hwang G. Y. (2015). Polygonum cuspidatum and its active components inhibit replication of the influenza virus through toll-like receptor 9-induced interferon beta expression. PLoS One 10 (2), e0117602. 10.1371/journal.pone.0117602 25658356 PMC4319845

[B31] Li N. Lu R. Yu Y. Lu Y. Huang L. Jin J. (2016). Protective effect of Periplaneta americana extract in ulcerative colitis rats induced by dinitrochlorobenzene and acetic acid. Pharm. Biol. 54 (11), 2560–2567. 10.3109/13880209.2016.1170862 27309769

[B32] Liu S. H. Chuang W. C. Lam W. Jiang Z. Cheng Y. C. (2015). Safety surveillance of traditional Chinese medicine: current and future. Drug Sa. 38 (2), 117–128. 10.1007/s40264-014-0250-z PMC4348117 25647717

[B33] Liu B. Li S. Sui X. Guo L. Liu X. Li H. (2018). Root Extract of Polygonum cuspidatum Siebold and Zucc. Ameliorates DSS-Induced Ulcerative Colitis by Affecting NF-kappaB Signaling Pathway in a Mouse Model via Synergistic Effects of Polydatin, Resveratrol, and Emodin. Front. Pharmacol. 9347, 347. 10.3389/phar.2018.00347 PMC5904535 29695964

[B34] Ma W. K. Li H. Dong C. L. He X. Guo C. R. Zhang C. . (2016). Palmatine rom Mahonia bealei attenuates gut tumorigenesis in ApcMin/+ mice via inhibition of inlammatory cytokines. Mol. Med. Rep. 14 (1), 491–498. 10.3892/mmr.2016.5285 27175745 PMC4918606

[B35] Ma H. Hu Z. C. Long Y. Cheng L. C. Zhao C. Y. Shao M. K. (2022). Tanshinone IIA Microemulsion Protects against Cerebral Ischemia Reperfusion Injury via Regulating H3K18ac and H4K8ac *In Vivo* and *In Vitro* . Am. J. Chin. Med. 50 (7), 1845–1868. 10.1142/S0192415X22500781 36185015

[B36] Ma X. Zheng C. Hu C. Rahman K. Qin L. (2011). The genus Desmodium (Fabaceae)-traditional uses in Chinese medicine, phytochemistry and pharmacology. J. Ethnopharmacol. 138 (2), 314–332. 10.1016/j.jep.2011.09.053 22004895

[B37] Megahed F. A. K. Zhou X. Sun P. (2020). The interactions between HBV and the innate immunity of hepatocytes. Viruses 12 (3), 285. 10.3390/v12030285 32151000 PMC7150781

[B38] MOFCOM (2022). Statistical analysis report on the operation of the drug distribution industry in 2021. Ministry of Commerce of the People’s Republic of China. Available at: http://www.mofcom.gov.cn/article/zwgk/gkfxbg/202209/20220903345957.shtml (Accessed: 2024).

[B39] Muanda F. N. Bouayed J. Djilani A. Yao C. Soulimani R. Dicko A. (2011). Chemical composition and, cellular evaluation of the antioxidant activity of desmodium adscendens leaves. Evid. Based Complement. Altern. Med. 2011, 620862. 10.1155/2011/620862 PMC2957201 20976084

[B40] Mussard E. Cesaro A. Lespessailles E. Legrain B. Berteina-Raboin S. Toumi H. (2019). Andrographolide, a natural antioxidant: an update. Antioxidants (Basel) 8 (12), 571. 10.3390/antiox8120571 31756965 PMC6943416

[B41] NATCM (2016). Law of the people’s republic of China on traditional chinese medicine. Natl. Adm. Traditional Chin. Med. Available at: http://www.natcm.gov.cn/ajiansi/zhengcewenjian/2018-03-24/2249.html (Accessed: 2024).

[B42] NMPA (2000). A notice on the rectification of traditional Chinese medicine health drugs. Natl. Med. Prod. Adm. Available at: https://www.nmpa.gov.cn/xxgk/gwj/gzwj/gzwjyp/20000307010101371.html (Accessed: 2024).

[B43] NMPA (2002). Notice on Unified Replacement and Standardization of Drug Approval Number Format, National Medical Products Administration. Available at: https://www.nmpa.gov.cn/xxgk/fgwj/gzwj/gzwjyp/20020128010101658.html (Accessed: 2024).

[B44] NMPA (2017a). Notice of the National Medical Product Administration of China on the Issuance of Technical Guidelines for the Naming of General Names of CCPPs (2017 No. 188). Natl. Med. Prod. Adm. Available at: https://www.nmpa.gov.cn/xxgk/ggtg/ypggtg/ypqtggtg/20171128180501239.html (Accessed: 2024).

[B45] NMPA (2017b). What is CCPP, national medical products administration. Available at: https://www.nmpa.gov.cn/xxgk/kpzhsh/kpzhshyp/20171024101101251.html (Accessed: 2024).

[B46] NMPA (2020). A Notice of the National Medical Products Administration on Issuing the Requirements for Classification and Application Materials of Traditional Chinese Medicine Registration (No. 68 of 2020). Natl. Med. Prod. Adm. Available at: https://www.nmpa.gov.cn/xxgk/ggtg/ypggtg/ypqtggtg/20200928164311143.html (Accessed: 2024).

[B47] NMPA (2023) “National drug coding standard code (as of June 30, 2023),” in National medical products administration. Available at: https://www.nmpa.gov.cn/zwfw/zwfwzxfw/zxfwsjxz/20230706145013131.html (Accessed: 2024).

[B48] Royal Botanic Gardens, Kew (2023). Medicinal plant names Services. Kew: Royal Botanic Gardens. Available at: https://mpns.science.kew.org/mpns-portal (Accessed 2024).

[B49] SAMR (2020). Measures for the administration of drug registration. State Administration for Market Regulation. Available at: https://www.samr.gov.cn/zw/zfxxgk/fdzdgknr/fgs/art/2023/art_3275cb2a929d4c34ac8c0421b2a9c257.html (Accessed: 2024).

[B50] Singh N. Krishnakumar S. Kanwar R. K. Cheung C. H. Kanwar J. R. (2015). Clinical aspects for survivin: a crucial molecule for targeting drug-resistant cancers. Drug Discov. Today 20 (5), 578–587. 10.1016/j.drudis.2014.11.013 25433305

[B51] Tao R. Lu K. Zong G. Xia Y. Han H. Zhao Y. (2023). Ginseng polysaccharides: potential antitumor agents. J. Ginseng Res. 47 (1), 9–22. 10.1016/j.jgr.2022.07.002 36644386 PMC9834022

[B52] TCM Clinical Evidence Database (2023). Randomized controlled trials involving CCPPs from 2020 to 2022, TCM Clinical Evidence Database. Available at: https://www.tcmevd.com/evidence/index (Accessed: 2024).

[B53] Thakur A. K. Rai G. Chatterjee S. S. Kumar V. (2016). Beneficial effects of an andrographis paniculata extract and andrographolide on cognitive functions in streptozotocin-induced diabetic rats. Pharm. Biol. 54 (9), 1528–1538. 10.3109/13880209.2015.1107107 26810454

[B54] The Beijing News (2023). “Innovation Report on China’s Traditional Chinese Medicine Industry: last year, the top 30 enterprises invested over 7.4 billion yuan,” in Research and development (The Beijing News). Available at: https://m.bjnews.com.cn/detail/1682052660169124.html (Accessed: 2024).

[B55] Wallock-Richards D. Doherty C. J. Doherty L. Clarke D. J. Place M. Govan J. R. (2014). Garlic revisited: antimicrobial activity of allicin-containing garlic extracts against Burkholderia cepacia complex. PLoS One 9 (12), e112726. 10.1371/journal.pone.0112726 25438250 PMC4249831

[B56] Wang M. R. Zhang X. J. Liu H. C. Ma W. D. Zhang M. L. Zhang Y. (2019). Matrine protects oligodendrocytes by inhibiting their apoptosis and enhancing mitochondrial autophagy. Brain Res. Bull. 153, 15330–15338. 10.1016/j.brainresbull.2019.08.006 31404585

[B57] Wang B. Zou F. Xin G. Xiang B. L. Zhao J. Q. Yuan S. F. (2023). Sodium tanshinone IIA sulphate inhibits angiogenesis in lung adenocarcinoma via mediation of miR-874/eEf-2K/TG2 axis. Pharm. Biol. 61 (1), 868–877. 10.1080/13880209.2023.2204879 37300283 PMC10259344

[B58] Wang D. Huang L. Chen S. (2013). Senecio scandens Buch.-Ham.: a review on its ethnopharmacology, phytochemistry, pharmacology, and toxicity. J. Ethnopharmacol. 149 (1), 1–23. 10.1016/j.jep.2013.05.048 23747644

[B59] Wikipedia (2024). List of patent medicines. Wikipedia. Available at: https://en.wikipedia.org/wiki/List_of_patent_medicines (Accessed: 2024).

[B60] Wu J. Xiao Q. Zhang N. Xue C. Leung A. W. Zhang H. (2016). Photodynamic action of palmatine hydrochloride on colon adenocarcinoma HT-29 cells. Photodiagnosis Photodyn. Ther. 155, 53–58. 10.1016/j.pdpdt.2016.05.005 27181460

[B61] Xu T. Wang Z. Lei T. Lv C. Wang J. Lu J. (2015). New flavonoid glycosides from Sedum aizoon L. Fitoterapia 1011, 125–132. 10.1016/j.itote.2014.12.014 25562804

[B62] Yang C. Mu Y. Li S. Zhang Y. Liu X. Li J. (2023). Tanshinone IIA: a Chinese herbal ingredient for the treatment of atherosclerosis. Front. Pharmacol. 14, 1321880. 10.3389/phar.2023.1321880 38108067 PMC10722201

[B63] Yue S. J. Liu J. Feng W. W. Zhang F. L. Chen J. X. Xin L. T. (2017). System pharmacology-based dissection of the synergistic mechanism of huangqi and huanglian for diabetes mellitus. ront. Pharmacol. 8694, 694. 10.3389/phar.2017.00694 PMC5633780 29051733

[B64] Yun J. Hwang J. S. Lee D. G. (2017). The antifungal activity of the peptide, periplanetasin-2, derived from American cockroach *Periplaneta americana* . Biochem. J. 474 (17), 3027–3043. 10.1042/BCJ20170461 28733329

[B65] Zeng H. Wang Y. Gu Y. Wang J. Zhang H. Gao H. (2019). Polydatin attenuates reactive oxygen species-induced airway remodeling by promoting Nrf2-mediated antioxidant signaling in asthma mouse model. Life Sci. 218, 21825–21830. 10.1016/j.ls.2018.08.013 30092299

[B66] Zhang M. Y. Yu Y. Y. Wang S. F. Zhang Q. Wu H. W. Wei J. Y. (2018). Cardiotoxicity evaluation of nine alkaloids from Rhizoma Coptis. Hum. Exp. Toxicol. 37 (2), 185–195. 10.1177/0960327117695633 29233041

[B67] Zhang X. Zhu Y. Duan W. Feng C. He X. (2015). Allicin induces apoptosis of the MGC-803 human gastric carcinoma cell line through the p38 mitogen-activated protein kinase/caspase-3 signaling pathway. Mol. Med. Rep. 11 (4), 2755–2760. 10.3892/mmr.2014.3109 25523417

[B68] Zhao X. Y. Zhang F. Pan W. Yang Y. F. Jiang X. Y. (2021). Clinical potentials of ginseng polysaccharide for treating gestational diabetes mellitus. World J. Clin. Cases 9 (19), 4959–4979. 10.12998/wjcc.v9.i19.4959 34307546 PMC8283579

[B69] Zhao Y. Yang A. Tu P. Hu Z. (2017). Anti-tumor effects of the American cockroach, *Periplaneta americana* . Chin. Med. 12, 26. 10.1186/s13020-017-0149-6 28919922 PMC5596864

[B70] Zhong J. Liu Z. Zhou X. Xu J. (2017). Synergic anti-pruritus mechanisms of action for the radix Sophorae flavescentis and fructus cnidii herbal pair. Molecules 22 (9), 1465. 10.3390/molecules22091465 28869563 PMC6151778

[B71] Zhou X. Pan Y. Wang Y. Wang B. Yan Y. Qu Y. (2021). Tanshinones induce tumor cell apoptosis via directly targeting FHIT. Sci. Rep. 11 (1), 12217. 10.1038/s41598-021-91708-z 34108553 PMC8190080

[B72] Zhu J. J. Yao S. Guo X. Yue B. S. Ma X. Y. Li J. (2018). Bioactivity-guided screening o wound-healing active constituents from American cockroach (*Periplaneta americana*). Molecules 23 (1), 101. 10.3390/molecules23010101 29361715 PMC6017267

[B73] Zhu Z. Z. Ma K. J. Ran X. Zhang H. Zheng C. J. Han T. (2011). Analgesic, anti-inflammatory and antipyretic activities of the petroleum ether fraction from the ethanol extract of Desmodium podocarpum. J. Ethnopharmacol. 133 (3), 1126–1131. 10.1016/j.jep.2010.11.042 21126565

