# Supplementary material for: Commercial Chinese polyherbal preparation: current status and future perspectives
Source: Front Pharmacol. 2024 Jul 24;15:1404259. doi: 10.3389/fphar.2024.1404259 (PMC11306874; doi:10.3389/fphar.2024.1404259)
Supplement: Supplementary file 2 [file DataSheet4.PDF]

Supplementary Table S2. CBDs with the Top 20 Highest Application Frequency among All CCPPs' Prescription

(According to the Treated Diseases with Deficiency Syndrome or Excess Syndrome)

Diseases with Deficiency Syndrome (2,221, 22.24%)		Diseases with Excess Syndrome (6,194, 62.03%)		No Obvious Tendency of Deficiency Syndrome or Excess Syndrome (1,571, 15.73%)	
CBDs	Application Frequency (Rate)	CBDs	Application Frequency (Rate)	CBDs	Application Frequency (Rate)
A. mongholicus	(690,31.07%)	*G. glabra*	(1350,21.80%)	*A. sinensis*	(354,22.53%)
A. sinensis	(575,25.89%)	*S. baicalensis*	(778,12.56%)	*A. mongholicus*	(351,22.34%)
W. cocos	(563,25.35%)	*A. sinensis*	(765,12.35%)	*G. glabra*	(314,19.99%)

L. barbarum	(500,22.51%)	*Borneolum syntheticum*	(761,12.29%)	*W. cocos*	(257,16.36%)
G. glabra	(478,21.52%)	*R. officinale*	(657,10.61%)	*A. macrocephala*	(254,16.17%)
R. glutinosa	(452,20.35%)	*C. tinctorius*	(603,9.74%)	*L. chuanxiong*	(246,15.66%)
P. ginseng	(449,20.22%)	*L. chuanxiong*	(578,9.33%)	*P. lactiflora*	(234,14.89%)
A. macrocephala	(415,18.69%)	*P. grandiflorus*	(557,8.99%)	*S. miltiorrhiza*	(232,14.77%)
C. pilosula	(401,18.05%)	*A. lappa Decne*	(518,8.36%)	*R. glutinosa*	(208,13.24%)

S. chinensis	(385,17.33%)	*C. reticulata*	(510,8.23%)	*C. pilosula*	(172,10.95%)
Dioscorea oppositifolia L. [Dioscoreaceae; Dioscoreae rhizoma]	(371,16.70%)	*L. japonica*	(481,7.77%)	*C. monogyna*	(160,10.18%)
R. glutinosa	(341,15.35%)	*S. miltiorrhiza*	(466,7.52%)	*P. ginseng*	(154,9.80%)
P. lactiflora	(334,15.04%)	*Mentha canadensis L.*	(465,7.51%)	*C. reticulata*	(152,9.68%)
E. brevicornu	(322,14.50%)	*Angelica dahurica* (Hoffm.) Benth. & Hook.f. ex Franch. & Sav. [Apiaceae; Angelicae dahuricae radix]	(441,7.12%)	*C. tinctorius*	(127,8.08%)
R. multiflora	(302,13.60%)	*P. cocos*	(441,7.12%)	*O. japonicus*	(118,7.51%)

C. australis	(289,13.01%)	*G. jasminoides*	(428,6.91%)	*D. oppositifolia L.*	(117,7.45%)
O. japonicus	(278,12.52%)	*F. suspensa*	(424,6.85%)	*P. notoginseng*	(116,7.38%)
Cervus nippon Temminck	(248,11.17%)	*B. chinense dc.*	(389,6.28%)	*L. barbarum*	(116,7.38%)
C. reticulata	(246,11.08%)	*P. notoginseng*	(387,6.25%)	*R. multiflora*	(112,7.13%)
C. deserticola	(229,10.31%)	*Paeonia lactiflora* Pall. [Paeoniaceae; Paeoniae radix rubra]	(386,6.23%)	*S. chinensis*	(112,7.13%)
