# Supplementary material for: Commercial Chinese polyherbal preparation: current status and future perspectives
Source: Front Pharmacol. 2024 Jul 24;15:1404259. doi: 10.3389/fphar.2024.1404259 (PMC11306874; doi:10.3389/fphar.2024.1404259)
Supplement: Supplementary file 3 [file DataSheet3.PDF]

Supplementary Table S1. CBDs with the Top 20 Highest Application Frequency among All CCPPs' Prescription

(According to the Treated Diseases Caused by Pathogenic Cold or Pathogenic Heat)

Diseases Caused by Pathogenic Heat (3,850, 38.55%)		Diseases Caused by Pathogenic Cold (1,305, 13.07%)		Diseases of No Obvious Cause of Cold and Heat (4,831, 48.38%)	
CBDs	Application Frequency (Rate)	CBDs	Application Frequency (Rate)	CBDs	Application Frequency (Rate)
Glycyrrhiza glabra L. [Fabaceae; Glycyrrhizae radix et rhizoma]	(910,23.64%)	*A. sinensis*	(337,25.82%)	*A. sinensis*	(1029,21.30%)
Scutellaria baicalensis Georgi [Lamiaceae; Scutellariae radix]	(711,18.47%)	*G. glabra*	(334,25.59%)	*G. glabra*	(898,18.59%)
Lonicera japonica Thunb. [Caprifoliaceae; Lonicerae japonicae flos]	(483,12.55%)	*Cinnamomum verum* J.Presl [Lauraceae; Cinnamomi cortex]	(274,21.00%)	*A. mongholicus*	(851,17.62%)

Rheum officinale Baill. [Polygonaceae; Rhei radix et rhizoma]	(472,12.26%)	*Epimedium brevicornu* Maxim. [Berberidaceae; Epimedii folium]	(229,17.55%)	*W. cocos*	(695,14.39%)
Dryobalanops aromatica C.F.Gaertn. [Dipterocarpaceae; Borneolum syntheticum]	(429,11.14%)	*W. cocos*	(228,17.47%)	*L. chuanxiong*	(655,13.56%)
Forsythia suspensa (Thunb.) Vahl [Oleaceae; Forsythiae fructus]	(387,10.05%)	*Astragalus mongholicus* Bunge [Fabaceae; Astragali radix]	(218,16.70%)	*A. macrocephala*	(596,12.34%)
Platycodon grandiflorus (Jacq.) A.DC. [Campanulaceae; Platycodonis radix]	(386,10.03%)	*Ligusticum chuanxiong* Hort. [Apiaceae; Chuanxiong rhizoma]	(199,15.25%)	*Salvia miltiorrhiza* Bunge [Lamiaceae; Salviae miltiorrhizae radix et rhizoma]	(588,12.17%)
Gardenia jasminoides J.Ellis [Rubiaceae; Gardeniae fructus]	(382,9.92%)	*Panax ginseng* C.A.Mey. [Araliaceae; Ginseng radix et rhizoma]	(185,14.18%)	*P. lactiflora*pall.	(536,11.10%)
Rehmannia glutinosa (Gaertn.) DC. [Orobanchaceae; Rehmanniae radix]	(369,9.58%)	*Lycium barbarum* L. [Solanaceae; Lycii fructus]	(179,13.72%)	*C. reticulata*	(524,10.85%)

W. cocos	(338,8.78%)	*Atractylodes macrocephala* Koidz. [Asteraceae; Atractylodis macrocephalae rhizoma]	(176,13.49%)	*Codonopsis pilosula* (Franch.) Nannf. [Campanulaceae; Codonopsis radix]	(488,10.10%)
Mentha canadensis L. [Lamiaceae; Menthae haplocalycis herba]	(334,8.68%)	*Rehmannia glutinosa* (Gaertn.) DC. [Orobanchaceae; Rehmanniae radix praeparata]	(166,12.72%)	*C. tinctorius*	(470,9.73%)
Coptis chinensis Franch. [Ranunculaceae; Coptidis rhizoma]	(333,8.65%)	*P. lactiflora*	(165,12.64%)	*P. ginseng*	(453,9.38%)
Angelica sinensis (Oliv.) Diels [Apiaceae; Angelicae sinensis radix]	(328,8.52%)	*Citrus reticulata* Blanco [Rutaceae; Citri reticulatae pericarpium]	(165,12.64%)	*Panax notoginseng* (Burkill) F.H.Chen [Araliaceae; Notoginseng radix et rhizoma]	(425,8.80%)
Gypsum Fibrosum [Mineral; Gypsum]	(316,8.21%)	*Achyranthes bidentata* Blume [Amaranthaceae; Achyranthis bidentatae radix]	(154,11.80%)	*R. glutinosa*	(418,8.65%)
Phellodendron chinense C.K.Schneid. [Rutaceae; Phellodendri chinensis cortex]	(310,8.05%)	*Carthamus tinctorius* L. [Asteraceae; Carthami flos]	(149,11.42%)	*L. barbarum*	(415,8.59%)

Bovis calculus	(304,7.90%)	*C. verum*	(148,11.34%)	*Aucklandia lappa* Decne [Asteraceae; Aucklandiae radix]	(409,8.47%)
Isatis tinctoria subsp. tinctoria [Brassicaceae; Isatidis radix]	(292,7.58%)	*Cistanche deserticola* Ma [Orobanchaceae; Cistanches herba]	(148,11.34%)	*Schisandra chinensis* (Turcz.) Baill. [Schisandraceae; Schisandrae chinensis fructus]	(389,8.05%)
Bupleurum chinense DC. [Apiaceae; Bupleuri radix]	(272,7.06%)	*Ephedra sinica* Stapf [Ephedraceae; Ephedrae herba]	(144,11.03%)	*Reynoutria multiflora* (Thunb.) Moldenke [Polygonaceae; Polygoni multiflori radix]	(385,7.97%)
Ophiopogon japonicus (Thunb.) Ker Gawl. [Asparagaceae; Ophiopogonis radix]	(264,6.86%)	*Cervi cornu pantotrichum*	(142,10.88%)	*R. glutinosa*	(373,7.72%)
Paeonia lactiflora Pall. [Paeoniaceae; Paeoniae radix alba]	(248,6.44%)	*Cuscuta australis* R.Br. [Convolvulaceae; Cuscutae semen]	(141,10.80%)	*Crataegus monogyna* Jacq. [Rosaceae; Crataegi fructus]	(369,7.64%)
