# Supplementary material for: Commercial Chinese polyherbal preparation: current status and future perspectives
Source: Front Pharmacol. 2024 Jul 24;15:1404259. doi: 10.3389/fphar.2024.1404259 (PMC11306874; doi:10.3389/fphar.2024.1404259)
Supplement: Supplementary file 5 [file DataSheet5.PDF]

Supplementary Table S3. CBDs with the Top 20 Highest Application Frequency among All CCPPs' Prescription

(According to the Treated Diseases with Exterior Syndrome or Interior Syndrome)

Diseases with Exterior Syndrome (1,349, 13.51%)		Diseases with Interior Syndrome (8,427, 84.39%)		Disease Involving Both Exterior and Interior(210, 2.10%)	
CBDs	Application Frequency (Rate)	CBDs	Application Frequency (Rate)	CBDs	Application Frequency (Rate)
G. glabra	(458,33.95%)	*G. glabra*	(1629,19.33%)	*G. glabra*	(55,26.19%)
P. grandiflorus	(377,27.95%)	*A. sinensis*	(1628,19.32%)	*C. reticulata*	(44,20.95%)
L. japonica	(264,19.57%)	*A. mongholicus*	(1242,14.74%)	*W. cocos*	(40,19.05%)

S. baicalensis	(257,19.05%)	*P. cocos*	(1121,13.30%)	*Magnolia officinalis* Rehder & E.H.Wilson [Magnoliaceae; Magnoliae officinalis cortex]	(38,18.10%)
Mentha canadensis L.	(237,17.57%)	*L. chuanxiong*	(961,11.40%)	*S. baicalensis*	(37,17.62%)
F. suspensa	(236,17.49%)	*P. lactiflora*	(888,10.54%)	*Mentha canadensis L.*	(35,16.67%)
E. sinica	(179,13.27%)	*S. miltiorrhiza*	(874,10.37%)	*A. dahurica*	(32,15.24%)
C. reticulata	(174,12.90%)	*A. macrocephala*	(834,9.90%)	*Atractylodes lancea* (Thunb.) DC. [Asteraceae; Atractylodis rhizoma]	(32,15.24%)
I. tinctoria	(158,11.71%)	*R. glutinosa*	(813,9.65%)	*Pinellia ternata* (Thunb.) Makino [Araceae; Pinelliae rhizoma]	(32,15.24%)

Prunus armeniaca L. [Rosaceae; Armeniacae semen amarum]	(152,11.27%)	*C. tinctorius*	(764,9.07%)	*Pogostemon cablin* (Blanco) Benth. [Lamiaceae; Pogostemonis herba]	(30,14.29%)
Gypsum Fibrosum	(149,11.05%)	*Borneolum syntheticum*	(735,8.72%)	*R. officinale*	(28,13.33%)
Saposhnikovia divaricata (Turcz. ex Ledeb.) Schischk. [Apiaceae; Saposhnikoviae radix]	(135,10.01%)	*P. ginseng*	(731,8.67%)	*A. sinensis*	(24,11.43%)
A. dahurica	(113,8.38%)	*A. lappa Decne*	(692,8.21%)	*S. divaricata*	(23,10.95%)
Dysphania ambrosioides (L.) Mosyakin & Clemants [Amaranthaceae; Schizonepetae herba]	(110,8.15%)	*C. reticulata*	(690,8.19%)	*A. macrocephala*	(23,10.95%)
Stemona tuberosa Lour. [Stemonaceae; Stemonae radix]	(106,7.86%)	*R. officinale*	(679,8.06%)	*P. grandiflorus*	(22,10.48%)

A. lancea	(105,7.78%)	*C. pilosula*	(675,8.01%)	*Borneolum syntheticum*	(21,10.00%)
B. chinense DC	(103,7.64%)	*L. barbarum*	(669,7.94%)	*L. chuanxiong*	(20,9.52%)
Kitagawia praeruptora (Dunn) Pimenov [Apiaceae; Peucedani radix]	(102,7.56%)	*S. baicalensis*	(608,7.21%)	*A. mongholicus*	(20,9.52%)
W. cocos	(100,7.41%)	*S. chinensis*	(608,7.21%)	*B. chinense DC*	(19,9.05%)
Borneolum syntheticum	(99,7.34%)	*R. glutinosa*	(603,7.16%)	*Coptis chinensis* Franch.	(18,8.57%)
